# Medico-Chirurgical Society of Louisville, Ky.

**Published:** 1881-05

**Authors:** 


### MEDICO CHIRURGICAL SOCIETY OF LOUISVILLE, KENTUCKY.

*The Treatment of Erysipelas.*—At the preceding meeting the subject of erysipelas was brought up by the report of a case in which tincture of aconite had been employed with good results. The discussion which followed its introduction led to its selection for discussion at this meeting.

Dr. Clements opened the discussion by reporting a case: An infant four months old was brought to me two weeks ago, having on the middle finger of the right hand a slight wound. The finger was swollen, and bore signs of erysipelas. I warned the mother of the danger, put the child upon the tinct. ferri chlor , and recommended her to poultice it and see me next day. She did not return for two days. At this time the swelling extended to the elbow. I ordered a simple cranberry poultice (altogether on empiricial grounds), and increased the tincture of chloride of iron. This failing to arrest it, and fever increasing, I ordered spiritus mindereri, with spirits of nitre, and the addition of tincture of aconite to the poultice. Its march was not at all impeded, apparently, as it soon reached the body, and taking a downward course, progressed to the umbilicus and below, when the opposite arm began to be likewise affected. Five days ago the child was opisthotonotic, and remained so for forty-eight hours. This yielded to full doses of bromide of potassium and gelsemium, but meantime the erysipelas progressed on toward the foot on the right side, and to near the elbow on the left arm. Day before yesterday, after an increase in the local trouble on the finger, there was a fresh lighting up of the disease at that point, and after a lively progress, followed by as rapid fading in its wage, it has now gone over all that portion of the body again, and is now half way between the umbilicus and the pubes, and still progressing. The temperature has ranged from 101° to 104°. It has been difficult to keep track of its pulse. It has nursed regularly, except during the manifestations of the meningeal trouble. The fact that the child is now living, with a good prospect of final recovery, is not less novel to me than the behavior of the disease.

Several authorities were then quoted by Dr. Clements to justify the means used locally with a view to check the progress of the disease.

Dr. F. Wilson said: Since the last meeting I have had a number of opportunities to test the tincture of aconite as a local application in erysipelas. I could not perceive that it lessened at all the burning pain, and after applying it three or four times I abandoned it, substituting the lead-and-opium wash. In the case to which I have reference, the temperature reached 105½°, and there was, for almost a week, low, muttering delirium. Yesterday the fever had subsided, and the appetite was returning, and every circumstance now indicated a speedy recovery.

Dr. Larrabee detailed two cases—one occurring in an infant six months old, treated by the internal administration of tinct. ferri, and the external application of a solution of sulphate of iron (3—O), cloths being wrung out of this solution and kept applied to the parts involved in the inflammation. This treatment, however, failed to arrest the progress of the disease, which ran its course unchecked, and ended in recovery after a great deal of trouble. Another case—that of an adult—treated by the internal administration of tinct. ferri chlor. and applications of iodine, continued its course until the solution above named superseded the iodine, when the disease was at once held in abeyance, and convalescence was quickly established.

In regard to the discussion of the subject, Dr. Larrabee said that, inasmuch as at the last meeting the sweeping assertion had been made that all local applications with a view to arrest the disease were of questionable advantage, and that, inasmuch as some surprise had been manifested that in this enlightened day physicians could be found who would advocate such means, he felt quite free to express surprise himself that any one could be found who would deny the efficacy of local applications. There was no work extant, said he, either ancient or modern, which was followed as a text-book, which did not endorse the application of astringent solutions for the arrest of inflammation in the early stage. That was one of the three principal indications for an astringent. Therefore, without the discussion of this point, it must be self-evident that the local treatment formed an important part of any rational mode of combatting erysipelas or any other inflammatory disease, provided it had not progressed to the formation of the products. Therefore he took it that the treatment of erysipelas should be both local and constitutional, and the local treatment should always be something more than palliative. As to arresting its progress at once, and for all, by the application of nitrate of silver, he had never been successful. He had tried it to his satisfaction in both hospital and private practice, and he now preferred the treatment of Virchow, as he had given it, because he found he could make the parts moist and comfortable, and in the vast majority of cases arrest the disease

Dr. Brandeis: I believe, sir, that erysipelas is entirely a toxæmia, from the fact that the slightest abrasion of the skin, placed under the condition of the impression by the poison, gives rise to erysipelas, while without this particular condition a large wound may exist without the appearance of the malady. Now, if we have such a science as pathology, we should make it an object to treat diseases according to that science. In the treatment of erysipelas, as many applications have been advocated for external use as there have been specifics recommended for whooping-cough. If it were an object to treat the disease with the view of subduing the local symptoms of inflammation, I would prefer the application of cold. But, if erysipelas is a toxæmia, it would appear more rational to treat local manifestations by palliative measures, using tonics, iron, and quinine internally, these being possibly supplemented by the hyposulphate of soda. But, as to the application of aconite, iron, or iodine, these have been used thousands of times without curtailing in any way the course of the disease.

Dr. Bailey: I would express sentiments somewhat in conformity to those of Dr. Brandeis, believing, as I do, this disease to be one of the blood, having as local and secondary manifestations this inflammation in the skin. I believe this to be true because we have constitutional symptoms twenty four, forty eight, and even a greater number of hours before local manifestations. When it occurs as the result of trauma, I do not believe the trauma alone adequate to its production. There is something beyond this and back of it that we do not comprehend, perhaps we never may, that renders this patient susceptible to erysipelas, and that insusceptible. Hence, I believe the disease to be first constitutional and afterwards local.

As to the management, I should say it ought to be largely expectant, not in a do-nothing sense, but I think it important not to meddle with a case until indications arise that demand treatment. The majority of cases are not modified by any treatment; it is a self-limited disease, and as for local applications, they appear to me to be worthless, from the fact that the processes they are intended to modify take place far beyond and out of reach of their influence. I cannot believe that local treatment shortens the disease one day. I have seen so many cases limited to such a small space without any treatment, that I am inclined to skepticism as to the result of all treatment in the disease. Patients often die for the want of support, and if I were the subject of it again, as I have been several times, I would watch for depression and endeaver to support myself, using any local applications solely with the view of promoting comfort.

Dr. Larrabee, our worthy President, has enunciated his views in no uncertain tones, and as we hold views so directly opposite, I feel it incumbent upon me to reply to his observations. I believe erysipelas is local before it is constitutional; and as he cites instances for maintaining the opposite view, in which the chill precedes local manifestations, so, in support of the opposite doctrine, I may appeal to the experience of every man to prove that we have local symptoms very frequently for hours before the patient feels the constitutional effects of the disease. The same thing is observed in idiopathic erysipelas that is seen in traumatism; it very rarely attacks a part unexposed. Let us suppose we have a patient brought into the surgical ward of the hospital with a clean, healthy looking wound, and that he is placed under circumstances favorable to the development of erysipelas. On the second day that wound will be dry, and the granulations, instead of being bathed in the laudable pus, and of that bright, healthy looking aspect, have become dry and sordid in appearance. The patient may have felt no change in himself up to this time, but a keen surgeon sees what that local condition will result in. Now, from the fact that in so called idiopathic cases the disease begins in an exposed part of the body, it is presumable that a solution of continuity existed there for the reception of the disease prior to the general symptoms of rigor and nausea.

As to the treatment, if, as the gentleman says, the disease has a tendency to rapid destruction and disintegration of blood-corpuscles, why should we wait for this actual destruction to take place? Would it not appear more rational to attempt to prevent this result than to attempt to restore them when once destroyed? I dare say he would not advocate that principle of treatment in diphtheria or any other disease in which it is an object to preserve the integrity of the blood.

It seems to me these points are well taken. Upon the decision as to the local or constitutional origin of the disease the treatment of each individual will be based.

